# Increased natural reproduction and genetic diversity one generation after cessation of a steelhead trout (*Oncorhynchus mykiss*) conservation hatchery program

**DOI:** 10.1371/journal.pone.0190799

**Published:** 2018-01-19

**Authors:** Barry A. Berejikian, Donald M. Van Doornik

**Affiliations:** 1 Environmental and Fisheries Sciences Division, Northwest Fisheries Science Center, National Marine Fisheries Service, National Oceanographic and Atmospheric Administration, Port Orchard, Washington, United States of America; 2 Conservation Biology Division, Northwest Fisheries Science Center, National Marine Fisheries Service, National Oceanographic and Atmospheric Administration, Port Orchard, Washington, United States of America; Ohio State University, UNITED STATES

## Abstract

Spatial and temporal fluctuations in productivity and abundance confound assessments of captive propagation programs aimed at recovery of Threatened and Endangered populations. We conducted a 17 year before-after-control-impact experiment to determine the effects of a captive rearing program for anadromous steelhead trout (*Oncorhynchus mykiss*) on a key indicator of natural spawner abundance (naturally produced nests or ‘redds’). The supplemented population exhibited a significant (2.6-fold) increase in redd abundance in the generation following supplementation. Four non-supplemented (control) populations monitored over the same 17 year period exhibited stable or decreasing trends in redd abundance. Expected heterozygosity in the supplemented population increased significantly. Allelic richness increased, but to a lesser (non-significant) degree. Estimates of the effective number of breeders increased from a harmonic mean of 24.4 in the generation before supplementation to 38.9 after supplementation. Several non-conventional aspects of the captive rearing program may have contributed to the positive response in the natural population.

## Introduction

Captive propagation programs have contributed to successful maintenance or amplification of some endangered populations [1,2]; however, the genetic risks associated with captive propagation [3,4], and challenges associated with reintroduction [5] raise the critical question of whether populations would fare better if left alone. Captive propagation efforts initially intended as temporary measures may become long-term endeavors if the factors causing declines are not adequately addressed [6]. Genetic risks may continue to accumulate throughout the duration of captivity [7], raising additional questions about when to terminate programs that have been successful at stemming declines in abundance. Population viability models can be used to predict population response to the termination of a captive propagation program, but predictions have rarely been evaluated because monitoring typically ends with the program [8]. Furthermore, influences of temporal environmental variability, natural fluctuations in abundance, and lack of monitored control populations confound assessments of effects on natural populations.

Application of captive propagation programs for anadromous salmonids has been remarkably controversial, and the focus of an incredible amount of empirical research [9]. Increasingly, salmonid “hatcheries” are being used for conservation [9] because of the continuing decline of both Atlantic (*Salmo salar*) and Pacific salmon (*Oncorhynchus* spp.) in many regions of world (e.g., [10,11,12]). Releases of hatchery-origin anadromous salmonids have clearly increased the abundance of adults reaching the spawning grounds [13,14,15]. Nevertheless, the conservation value of salmon hatcheries remains questionable because of evidence that hatchery populations experience altered selection pressures [16], suffer reduced fitness in the natural environment [17,18], exhibit reduced genetic diversity [19] and altered patterns of gene expression [20], and potentially impact the ecology of natural populations [21].

Some recent efforts have prevented extinction and maintained a large portion of genetic diversity from the founder population through carefully planned breeding protocols [22] and are trending towards the re-establishment of naturally self-sustaining populations [14]. Nevertheless, quantifying effects of programs on important natural population viability parameters remains a critical challenge. There are a few examples of ‘unplanned experiments’ that have assessed the relationships between large-scale hatchery programs and the productivity of natural populations after hatchery releases had been discontinued. Climate and density-dependent effects of hatcheries influenced coho salmon (*O*. *kisutch*) productivity on the Oregon Coast [23]. The densities of naturally produced Chinook salmon (*O*. *tshawytscha*) in streams receiving hatchery fish increased slightly (about 8%) relative to non-supplemented populations in the Snake River Basin [24]. In these and similar studies, lack of pre-supplementation data precludes before-after-control-impact (BACI) assessments of abundance, genetic diversity, and other program effects on natural populations. Efforts to quantify effects of hatchery programs can be obscured by spatial and temporal variability in freshwater environmental conditions or ocean productivity than can occur on annual or decadal time scales [24,25] and overwhelm signals caused by a hatchery program(s).

Declines in abundance of the Puget Sound steelhead trout (*O*. *mykiss*) Distinct Population Segment began in the late 1980s, followed by listing as Threatened under the US Endangered Species Act in 2007 (US Federal Register / Vol. 72, No. 91). Here, we assess the contribution of a conservation hatchery program to the abundance of redds (compilation of gravel nests constructed by females) created in the natural environment and effects on measures of genetic diversity in the generation after supplementation. The hatchery program evaluated here was different than most. Eyed embryos produced by naturally spawning, natural-origin steelhead were collected and transported to a hatchery where they were incubated, reared and released at two different life-history stages (smolt and adult). Effects on redd abundance and genetic diversity during supplemention (i.e., when hatchery-origin fish were spawning naturally) have been previously documented [15,26]. The BACI assessment was started prior to the hatchery program, includes multiple non-supplemented populations to incorporate interannual variability in freshwater and marine productivity, and by quantifying responses in the post-supplementation generation, provides information on the impacts of the hatchery program on the natural population.

## Methods

### Study area

**Fig 1:**
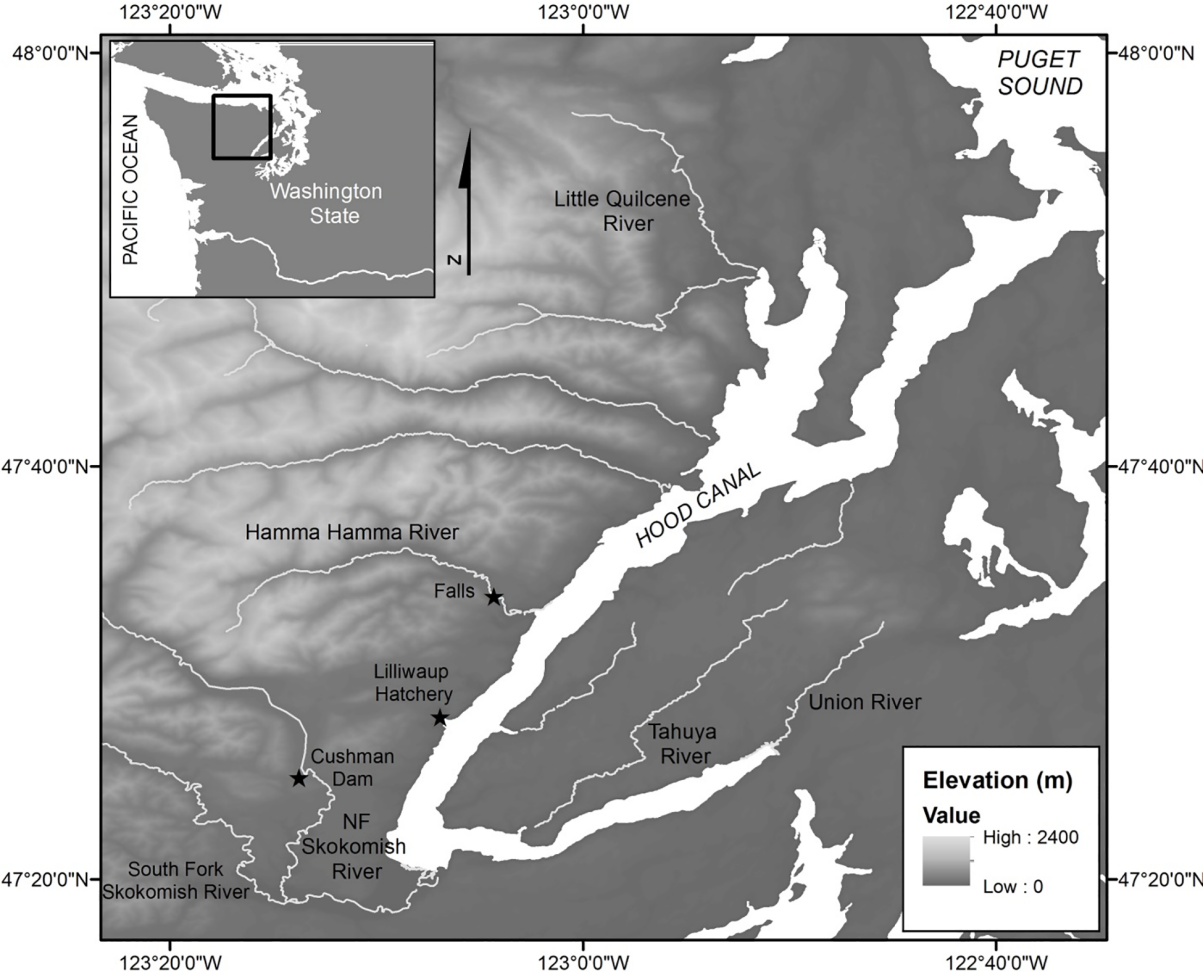
Map of the study area. Locations of the supplemented population (Hamma Hamma), the four non-supplemented populations (Tahuya, Little Quilcene, Union, and North Fork Skokomish), and the location of the captive rearing facility.

Hood Canal is an 80-km long fjord that is part of Puget Sound in western Washington State. The supplemented Hamma Hamma River watershed encompasses 137 km^2^ and flows into Hood Canal near the town of Eldon, WA (Fig 1). Anadromous fish passage is limited to the lower 3.6 km of the 29-km mainstem (Fig 1). Two non-supplemented populations inhabit the North Fork Skokomish River and Little Quilcene River, both of which also originate from the eastern slopes of the Olympic Mountains (Fig 1). The two additional non-supplemented streams discharge from the Kitsap Peninsula into the eastern side of Hood Canal (Tahuya River, Union River; Fig 1).

There are no reports of hatchery-origin steelhead smolts being released into the North Fork Skokomish or Little Quilcene Rivers before or during the study period. Steelhead smolts from a non-local, long-term hatchery broodstock were released into the Tahuya and Union Rivers in most years from the 1950s through 1993 [27], but not since. A genetic analyses conducted on the Tahuya population from 2007 to 2011 indicated that the Tahuya River natural population shows no evidence of introgression from the non-local hatchery stock; no genetic data are available from the Union River [26,27].

### Embryo collections, culture and release

The captive component of the Hamma Hamma population was established by hydraulic removal of eyed steelhead embryos in spring and summer [28]. A portion of the fish were reared for approximately two years and released at the smolt stage (seaward migrant) and are hereafter referred to as the smolt-release group (SRG; S1 Table). In some years, a portion of the SRG was retained at the Lilliwaup Hatchery for an additional two to three years and was released into the Hamma Hamma River near the time of final sexual maturation. These fish were referred to as the adult release group (ARG; S1 Table). Age-2 smolts were released during the first week of May each year beginning in 2000, and the ARG fish were released between 12 February and 6 March (depending on the year) to coincide with the onset of ovulation of captively reared females. Details on rearing practices and the body size of released SRG and ARG groups can be found in [15].

### Redd abundance

Steelhead females construct and deposit eggs into a series of nests, which form a contiguous ‘redd’. Each redd may contain up to eight individual nests [29]. Redds were identified as areas of excavated gravel, usually with a clear, crescent shaped ‘tail-spill’ at the downstream end and a depression or ‘pit’ at the upstream end [30]. Each female typically constructs one or two redds, and three independent studies suggest that females construct between 1.4 and 1.67 redds per female [29,30,31]. Redd surveys were conducted in the Hamma Hamma River and four non-supplemented rivers throughout Hood Canal (North Fork Skokomish, Little Quilcene, Tahuya, and Union; Fig 1) jointly by NOAA Fisheries, Washington Department of Fish and Wildlife, Skokomish Natural Resources Department, Long Live the Kings and the Hood Canal Salmon Enhancement Group. Redd counts in the Hamma Hamma River strongly correlate with visual estimates of abundance based on snorkel counts [15] and provide a measure more closely estimating reproductive output of a population because it incorporates pre-spawning mortality in freshwater. Redd counts are subject to annual variability in flow and visibility conditions, hence the incorporation of monitored control (non-supplemented) populations. The assumption is that the before-after-control-impact (BACI) analysis (see below) is not biased by any potential interaction between period (before or after supplementation) and population category (supplemented or control) in the ability to count redds.

Redd counts were analyzed from three periods (pre-supplementation: 1998–2001, during supplementation: 2002–2009, and post supplementation: 2010–2015). Surveys were conducted over nearly the entirety of each stream accessible to adult steelhead trout and encompassed the entire spawning period. The goal was to conduct a complete survey of each river approximately every 7 to 10 days, and the actual survey frequency depended on river depth and turbidity, which affect access and visibility. Surveys on any given day may have included the entire stream or some designated portion (reach) of the river. Each new observed redd was marked by attaching surveyors tape to riparian vegetation on the stream bank and recording the date each redd was observed to avoid re-counting the same redd on a subsequent survey. A study of “redd life” in Western Washington streams of similar size estimated that redds were visible for an average of 24 days [32], and no relationship was found between survey frequencies and redd counts in the Hamma Hamma River and Tahuya River in a previous study [15]. Nevertheless, to determine whether variation survey intensity varied over time and might bias redd count data, we quantified the annual amount of ‘effort’ expended on surveys in each river by calculating both the number of different days in which a partial or complete survey was conducted and the total length of stream surveyed (sum of all individual partial and complete surveys).

A total of 30 adult steelhead (18 natural origin and 12 returning smolt-released hatchery fish) captured by hook-and-line during the spawning seasons from 2002–2006 provided age-at-maturity data. Mean ocean age was 1.9 years (23.3% 1-ocean, 63% 2-ocean and 13.3% 3-ocean). Thus, for the purposes of comparing redd abundance over the three phases, we assumed a two-year ocean residence. We therefore considered 2002 as the first year in which hatchery fish were spawning naturally and 2009 as the last year. These years also correspond to the first and last years of the ARG releases.

We used a before-after-control-impact (BACI) analysis of variance (ANOVA) to test the effect of the captive rearing program on the abundance of redds. The same model was used to test for variation in two measures of redd survey effort (total distance surveyed and number of different days surveyed) to assess the possibility of unintentional bias in effort over time. The analyses were asymmetrical because there was only one impacted population and multiple control populations [33]. In this analysis, individual years were used as replicate measures within each period (before vs after). There were not replicate measures within each stream in a given year because repeated surveys were used to determine a single estimate for each population in each year. Count data were log transformed to improve normality and heteroscedasticity (there were no zero counts in any stream in any year). Alternative approaches, such as quasi-Poisson and negative binomial models, have been suggested [34]; however, others have more recently indicated that linear models perform well, may be less powerful, and provide control of type 1 error rates [35].

The ANOVA model took the form:

Y=μ+Ci+Bj+P(C)ik+T(B)jl+CBij+Eijkl Eq. 1

where: μ = grand mean, C_*i*_ = Category (supplemented or non-supplemented), B_j_ = Period (before or after), P(C)_ik_ = Population nested within Category, T(B)_*jl*_ = Year nested within Period, CB_*ij*_ = Category x Period interaction, and E_*ijkl*_ = Error term. A significant Category x Period interaction would indicate a differential response of supplemented and non-supplemented populations before and after supplementation for a given response variable. No redd abundance data were available for the Little Quilcene River in 1998. We used the expectation-maximization (EM) algorithm in Systat (V. 13) to generate a maximum likelihood estimate of the one missing redd count data point from the Little Quilcene River and two missing data points for survey effort (one in the Hamma Hamma River and one in the North Fork Skokomish River)

### Genetic diversity

Measures of genetic diversity were determined from juvenile *O*. *mykiss* samples collected in the Hamma Hamma River from 1999 through 2015 at two different time periods. In April and May each year *O*. *mykiss* smolts (silvery appearance, black fin margins) were collected in a rotary screw trap located less than 0.5 km upstream from the tidally influenced portion of the Hamma Hamma River. During summer months, parr less than 170 mm were collected by hook-and-line from throughout the anadromous-accessible portion of the Hamma Hamma River. A total of 667 parr and 305 smolts were assigned to an individual brood year based on their scale age and were analyzed for measures of genetic diversity. For each sample, DNA was extracted from a small piece of ethanol-preserved fin tissue and genotyped for 15 microsatellite DNA loci (see [36]. Appropriate scientific collection permits were obtained from the Washington Department of Fish and Wildlife.

The study plan and animal care procedures were approved by the NOAA Fisheries Northwest Fisheries Science Center. NOAA Fisheries also approved a Hatchery and Genetic Monitoring Plan for this study, which satisfies the criteria of the 4(d) rule under the Endangered Species Act. Fish were held for a minimal amount of time before tagging, anesthetized fully (80 mg/L Tricane Methanesulfonate), and allowed to completely recover before release. No tagged parr or smolts perished before release as a result of the sampling performed in this study, and all appeared to be alert, behaving normally, and in good condition upon release.

Three indicators of genetic diversity were estimated: expected heterozygosity, allelic richness, and the effective number of breeders (a measure of the effective population size). Expected heterozygosity was estimated within a sample using the program GENALEX 6.5 [37]. The program HP-RARE [38] was used to calculate allelic richness, which is a measure of within sample variability that takes sample size into account. Differences in these measures between pre- and post-supplementation samples were tested for significance with Wilcoxon signed-rank tests. Estimates of the effective number of breeders were made with the program NeEstimator [39], making use of the linkage disequilibrium method [40].

## Results

### Redd abundance

**Fig 2:**
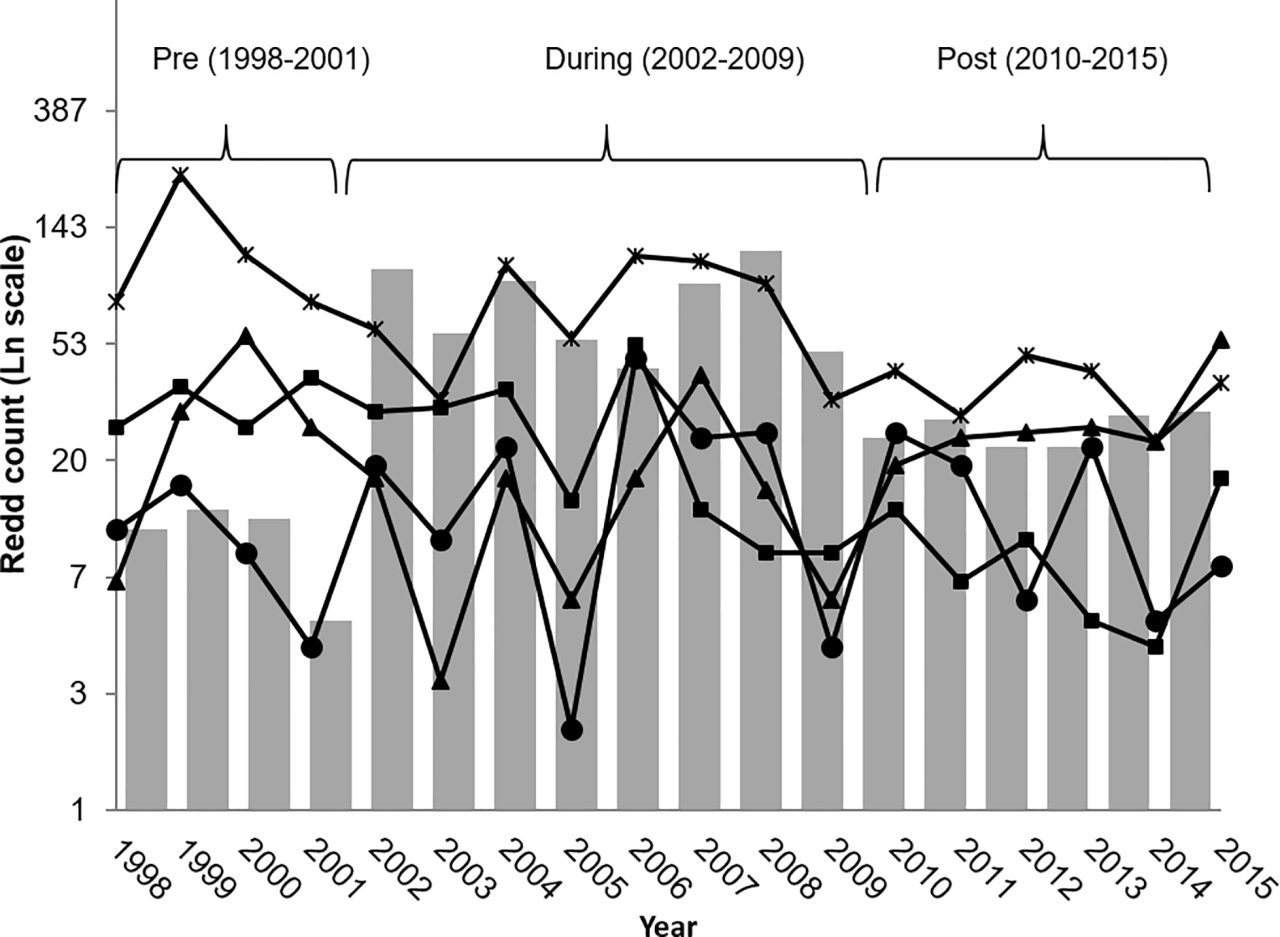
Annual redd counts in supplemented and non-supplemented populations. Redd counts in the supplemented population (Hamma Hamma River, grey bars) and four non-supplemented populations (Tahuya River, ∗; Little Quilcene River, ●; Union River, ■; and North Fork Skokomish River,▲) before, during and after supplementation.

A significant interaction between study period (before vs after) and category (supplemented vs non-supplemented) indicated a positive response to supplementation in the Hamma Hamma population (F_1,35_ = 12.705, P < 0.001; Table 1). The number of redds in the supplemented Hamma Hamma population increased from a mean of 10 redds pre-supplementation to 26 redds post-supplementation (Fig 2). Mean redd abundance in the non-supplemented populations either increased to a lesser extent (Little Quilcene: before = 10, after = 14), remained fairly stable (North Fork Skokomish: before = 30, after = 29) or declined substantially in the Tahuya River (before = 123, after = 37) and Union River (before = 31, after = 9; Fig 2). Redd survey effort differed significantly among the five rivers both in terms of the total number of days surveyed (F_3,35_ = 10.926, P < 0.001) and the total distance surveyed (F_3,35_ = 5.601, P = 0.003; S2 Table). However, there was no change in the survey effort in the two periods for either total distance surveyed (F_1,35_ = 0.924, P = 0.343) or frequency (F_1,35_ = 2.417, P = 0.129), and there was no interaction between period and category in either measure of survey effort (distance: F_1,35_ = 2.083, P = 0.158; frequency: F_1,35_ = 2.023, P = 0.164; S2 Table).

**Table 1:** Results of the test for the effects of supplementation on redd abundance. ANOVA results testing the effects of the conservation hatchery program on the abundance of redds. The main effects were category (supplemented or not) and period (before or after supplementation). The response variable of interest was the interaction between period and category. Significant effects (P <0.05) shown in bold.

Source of variation	df	MS	F-Ratio	p-Value
CATEGORY	1	1.026	3.015	0.091
PERIOD	1	0.402	1.181	0.285
**PERIOD x CATEGORY**	**1**	**4.325**	**12.705**	**0.001**
**POPULATION(CATEGORY)**	**3**	**5.476**	**16.086**	**0.000**
YEAR(PERIOD)	8	0.400	1.174	0.342
Error	35	0.340		

### Genetic diversity

**Fig 3:**
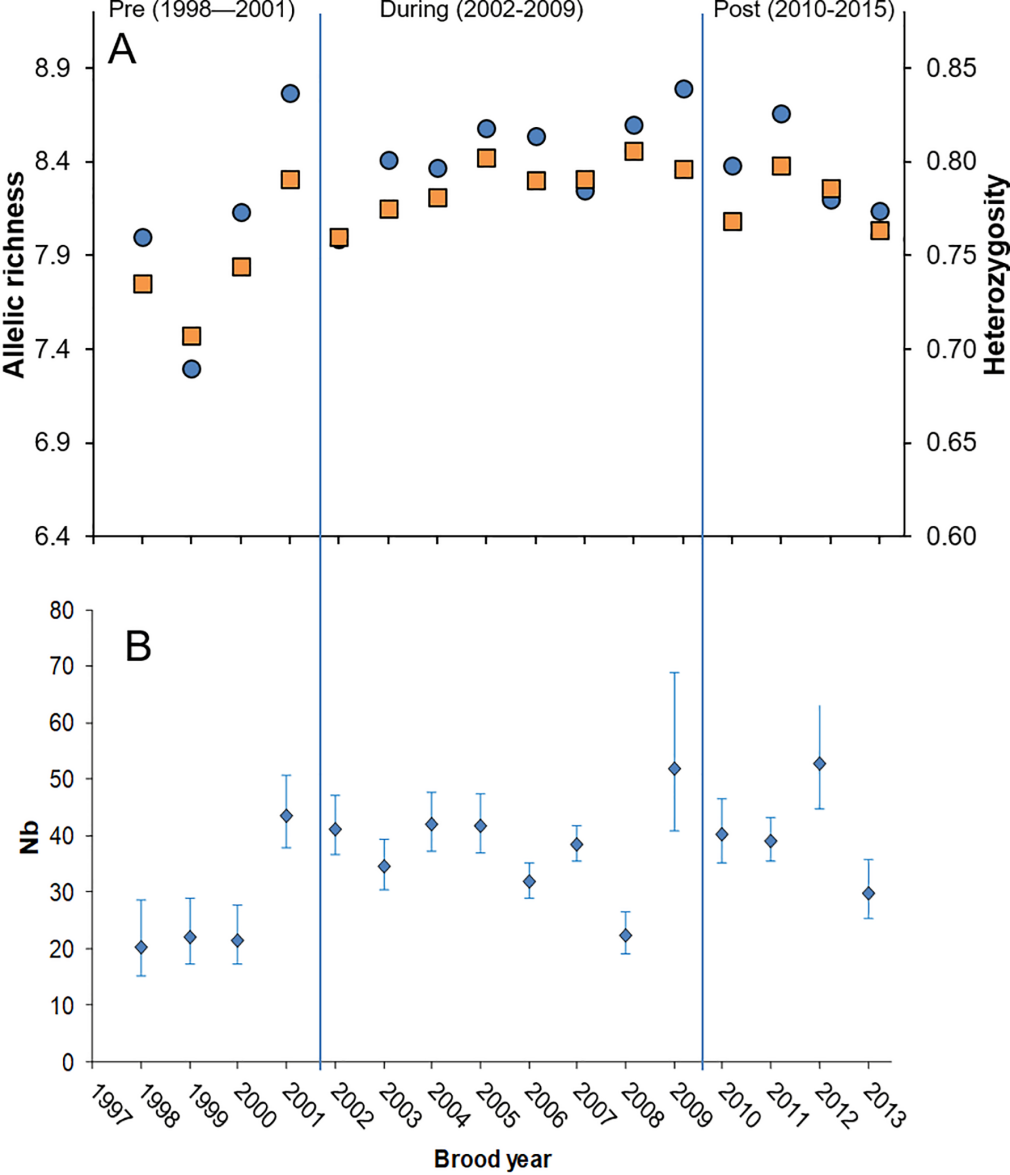
Measures of genetic diversity in the Hamma Hamma River. (A) Values of allelic richness (circles) and expected heterozygosity (squares) from Hamma Hamma River juvenile *O*. *mykiss* samples produced pre-, during-, and post-supplementation, and (B) estimates of the effective number of breeders (*N*_*b*_), with 95% confidence intervals, for Hamma Hamma River *O*. *mykiss* brood years representing pre-, during-, and post-supplementation periods.

Measures of expected heterozygosity and allelic richness both showed an increasing trend from pre- to post-supplementation samples (Fig 3A). The increase was significant for expected heterozygosity (*z* = -2.54, *P* = 0.011), but not quite for allelic richness (*z* = -1.69, *P* = 0.091). Similarly, estimates of effective population size increased (Fig 3B), with the harmonic mean of the post-supplementation samples (38.9) being greater than that of the pre-supplementation samples (24.4). Brood year 2001 had estimates of allelic richness and effective population size that were as high as later brood years, despite the fact that it was the year we observed the lowest number of redds. A low number of anadromous males may have allowed greater contributions from resident males successfully spawning with anadromous females introducing novel alleles into the anadromous population.

## Discussion

The potential for captive propagation programs to reduce extinction risk for anadromous salmonid populations has been highly controversial [41,42], and has raised important questions about impacts to natural populations from both genetic and ecological mechanisms [9,16,43]. In the Hamma Hamma River, the response to releases of captively reared juveniles and adults was substantial and immediate [18]. Although the number of redds was lower after supplementation than during (when hatchery-origin fish were spawning), the number of redds constructed in the Hamma Hamma River after supplementation was approximately 2.6 times greater than before supplementation. In contrast, the redd abundance in the four non-supplemented rivers had either declined or remained about the same, suggesting that the increases were related to the captive rearing program. The increase in reproductive output in the Hamma Hamma River was accompanied by increases in *N*_*b*_ and increased levels of heterozygosity, suggesting that overall genetic diversity was not compromised by the removal of naturally spawned embryos, captive rearing, and reintroduction, and may have increased. Any potential reduction in the genetic diversity of the Hamma Hamma River population caused by supplementation efforts might have been offset by the input of novel genes from strays from nearby populations. However, increased abundance during supplementation, stable or declining neighboring populations, and strong genetic structure among individual populations [34] suggest straying was probably very low during the study.

Multi-faceted recovery programs that include captive propagation, habitat restoration, harvest reductions, and changes in other management regimes have led to interim success in increasing population abundance [14,44]. For the Hamma Hamma steelhead population, the captive rearing program has been the primary change directly affecting the population over the timeframe of this study. Adult steelhead harvest had been discontinued on all populations before the study began, with the exception of a subsistence fishery in the mainstem Skokomish River. The Hamma Hamma River has not been the subject of any habitat restoration projects and the anadromous-accessible portion of the watershed continues to be privately managed in part for periodic timber harvest. Previous work in the Hamma Hamma River indicated that the captive rearing program caused a shift in the proportion of *O*. *mykiss* juveniles that were offspring of the anadromous form, largely attributable to F1 offspring from the ARG releases [27]. In the Hamma Hamma River and other Hood Canal *O*. *mykiss* populations, female steelhead offspring are less likely to mature in freshwater and more likely to smolt and produce anadromous adults [45] than offspring of resident females. Thus, any increase in proportion of juveniles from steelhead (including ARG) females, even under density-dependent freshwater growth and survival conditions, could have led to the increases in smolt production and redd production post-supplementation. Still, the Hamma Hamma steelhead population is fairly small, and historic harvest records indicate that the population provided an annual sport harvest of nearly 100 steelhead per year until a regional decline in species abundance in the early 1990s. Elevated mortality during the first three weeks after marine entry may be currently limiting productivity of the Hamma Hamma and other Hood Canal populations, which exhibit similar migratory behavior and survival patterns through Hood Canal, Admiralty Inlet, and Strait of Juan de Fuca to the Pacific Ocean [46,47].

This captive rearing program was intentionally designed and operated to alter some of the potential mechanisms that can negatively affect artificially propagated salmon and steelhead populations. Typical salmon and steelhead hatcheries collect sexually maturing adults and spawn them artificially, which removes any natural selection during reproduction and the opportunity for at least some of their gametes to contribute to natural reproduction. Conventional hatchery programs for salmon designed to produce fish for harvest (not conservation) have been associated with reductions in the *N*_*e*_*/N* ratio and accumulation of inbreeding [9]. Hydraulic embryo collections in the Hamma Hamma project came after natural and sexual selection had occurred in the natural environment, and small portions of a larger number of families were brought into captivity than would have been possible by collecting and spawning adults [28]. Fish were reared at low densities [48], on a restricted ration, and the SRG released at a natural smolt age (age-2), lessening the opportunity for size-selective mortality after release [16]. Furthermore, high survival rates in captivity provided amplification of the adult (ARG) population. Snorkel surveys and spawning observations conducted during the first portion of the ‘during supplementation’ phase (2002 and 2005) indicated that ARG females released in those years constructed an annual average of 48% of the observed redds [15]; redd construction from SRG and wild females constituted the remainder [15]. These and other types of hatchery strategies may hold promise for conservation programs where the emphasis lies with maintaining overall and adaptive genetic diversity and minimizing impacts (direct and indirect) to the natural populations.

There are critical determinations to be made about whether the risks of intervening with artificial propagation outweigh genetic risks that increase in small and declining populations faced with possible extirpation [49]. The genetic diversity measures we calculated are consistent with a population whose effective population size increased with the increase in spawners, which is consistent with theoretical expectations that effective size will increase with census size [50]. Other factors may have contributed to the increase in N_*b*_ post-supplementation: the embryo collection procedure incorporated a larger number of natural-origin parents than in typical hatchery programs where individual pairs are artificially spawned [28], and by removing only a portion of a female’s offspring, embryos not removed from sampled redds [15,28] still had the opportunity to contribute to natural production. The significant increase in heterozygosity post-supplementation may have resulted from increased spawning opportunities for resident (non-anadromous) males, which appear to contribute to genetic diversity by spawning with anadromous females [31]. The resident and anadromous components of this population show evidence of partial reproductive isolation and genetic differentiation [27,51]. An increased contribution from resident males, opportunistically spawning with the increased number of anadromous females released from the captive rearing program, may have increased diversity measures in a manner similar to the effects of immigration in small natural populations [52,53] by introducing novel alleles into the population. Studies of two other recent steelhead hatchery programs that spawn natural-origin adults as part of the hatchery broodstock have shown that the hatchery populations had a lower number of effective breeders and reduced allelic richness compared to their respective wild founding populations; however both measures of diversity either remained stable [19] or increased in the wild population [18] during supplementation (i.e., after returning hatchery fish began contributing to natural production). Neither study included post-supplementation data.

Conservation programs involving captive propagation have been increasing worldwide as species decline from a number of causes. Novel husbandry approaches designed to avoid potentially harmful practices hold some promise for short term interventions aimed at stemming declines or increasing the viability factors for depressed populations. Rearing strategies for numerous taxa are being developed and tested (e.g., [54,55]), and such efforts will be increasingly important as threats to natural populations increase. Longer term captive propagation programs maintaining abundance of species threatened with extinction may carry negative genetic consequences that increase with duration in captivity [7].

## Supporting information

S1 Table The number of redds from which embryos were collected, total number of embryos collected, number of juveniles released, and number of sexually maturing adult female steelhead released (males in parentheses) at age-4 or age-5 from the corresponding brood years. No embryo collections were attempted in 2002 or 2004 because of the large number of redds constructed in those years.
(DOCX)

S2 Table The mean (+/- sd) annual distance surveyed (km) and frequency of surveys conducted in the supplemented river (Hamma Hamma) and the four non-supplemented rivers (Tahuya, Little Quilcene, and Union, and North Fork Skokomish) before (1998–2001) and after (2010–2015) supplementation. (DOCX)
