# Supplementary material for: Increased natural reproduction and genetic diversity one generation after cessation of a steelhead trout (Oncorhynchus mykiss) conservation hatchery program
Source: PLoS One. 2018 Jan 19;13(1):e0190799. doi: 10.1371/journal.pone.0190799 (PMC5774695; doi:10.1371/journal.pone.0190799)
Supplement: S1 Table — No embryo collections were attempted in 2002 or 2004 because of the large number of redds constructed in those years. (DOCX) [file pone.0190799.s001.docx]

| Brood year | Redds sampled | Embryos Collected | Juveniles released | Adults released  Age-4 Age-5 | |
| --- | --- | --- | --- | --- | --- |
| 1998 | 8 | 4,683 | 3,235 | 81 (116) | 2 (2) |
| 1999 | 6 | 2,588 | 1,802 |  |  |
| 2000 | 7 | 1,622 | 1,090 | 35 (41) | 10 (15) |
| 2001 | 4 | 2,000 | 1,454 |  |  |
| 2003 | 23 | 4,105 | 2,043 | 58 (80) | 60 (77) |
| 2005 | 16 | 4,723 | 2,080 |  |  |
