# Supplementary material for: Increased natural reproduction and genetic diversity one generation after cessation of a steelhead trout (Oncorhynchus mykiss) conservation hatchery program
Source: PLoS One. 2018 Jan 19;13(1):e0190799. doi: 10.1371/journal.pone.0190799 (PMC5774695; doi:10.1371/journal.pone.0190799)
Supplement: S2 Table — (DOCX) [file pone.0190799.s002.docx]

|  | Frequency | | Distance | |
| --- | --- | --- | --- | --- |
|  | Before | After | Before | After |
| Hamma Hamma | 12 (1.2) | 13 (2.0) | 33.7 (1.2) | 31.7 (7.5) |
| Little Quilcene | 4 (2.1) | 9 (2.2) | 23.4 (13.5) | 67.8 (24.9) |
| NF Skokomish | 6 (0.6) | 22 (13.9) | 25.4 (5.2) | 75.4 (35.4) |
| Union | 7 (1.7) | 10 (2.8) | 49.4 (6.1) | 34.5 (9.4) |
| Tahuya | 16 (3.0) | 14 (2.4) | 124.9 (16.3) | 116.1 (26.9) |
